# Mind wandering perspective on attention-deficit/hyperactivity disorder

**DOI:** 10.1016/j.neubiorev.2018.07.010

**Published:** 2018-09

**Authors:** Natali S. Bozhilova, Giorgia Michelini, Jonna Kuntsi, Philip Asherson

**Affiliations:** King’s College London, Social, Genetic and Developmental Psychiatry Centre, Institute of Psychiatry, Psychology and Neuroscience, Denmark Hill, De Crespigny Park, SE5 8AF, United Kingdom

**Keywords:** ADHD, Mind wandering, Default mode network, Executive control network, Theory

## Abstract

Attention-Deficit/Hyperactivity Disorder (ADHD) is a common neurodevelopmental disorder associated with a range of mental health, neurocognitive and functional problems. Although the diagnosis is based on descriptions of behaviour, individuals with ADHD characteristically describe excessive spontaneous mind wandering (MW). MW in individuals with ADHD reflects constant mental activity which lacks topic stability and content consistency. Based on this review of the neural correlates of ADHD and MW, we outline a new perspective on ADHD: the MW hypothesis. We propose that altered deactivation of the default mode network, and dysfunctional interaction with the executive control network, leads to excessive and spontaneous MW, which underpins symptoms and impairments of ADHD. We highlight that processes linked to the normal neural regulation of MW (context regulation, sensory decoupling, salience thresholds) are deficient in ADHD. MW-related measures could serve as markers of the disease process, as MW can be experimentally manipulated, as well as measured using rating scales, and experience sampling during both cognitive tasks and daily life. MW may therefore be a potential endophenotype.

## Highlights

• Excessive, spontaneous mind wandering is associated with attention deficit hyperactivity disorder (ADHD).
• Deficient regulation of the default mode network in ADHD might lead to this type of mind wandering.
• This neural dysregulation might also underpin inattention and deficient cognitive performance.
• Converging evidence draws parallels between regulatory processes of mind wandering and deficient regulation in ADHD.

## 1 Introduction

Attention-Deficit/Hyperactivity Disorder (ADHD) is a common neurodevelopmental disorder affecting 5–6% of children and 3–4% of adults worldwide (Fayyad et al., 2007; Polanczyk et al., 2007). ADHD is characterised by developmentally inappropriate and impairing levels of inattentive, hyperactive and impulsive behaviours. The disorder is often accompanied by emotional lability (Skirrow et al., 2009), cognitive performance deficits (Banaschewski et al., 2012; Kofler et al., 2013) and mental health problems including anxiety, mood, personality and substance use disorders (Fayyad et al., 2007). ADHD is further linked to detrimental outcomes including educational and occupational failure, transport accidents with increased mortality (Kooij et al., 2010; Asherson et al., 2016) and criminal behaviour (Lichtenstein and Larsson, 2013).

Despite considerable progress in understanding the symptoms and impairments of ADHD and the availability of effective treatments (NICE, 2008), key clinical issues remain to be resolved. ADHD, particularly in adults, remains a disorder that often goes undiagnosed and untreated. One explanation is diagnostic uncertainty due to the high rates of psychiatric comorbidity and overlap of ADHD symptoms with other common mental health disorders (Asherson et al., 2016). The diagnosis also relies on subjective reports of symptoms and behaviours, leading to both under and over reporting of symptoms (Barkley et al., 2002; Du Rietz et al., 2016; Faraone and Biederman, 2016). Another problem is that current medications provide short-term control of ADHD symptoms, but do not bring about longer-term symptom remission, or adequate control of symptoms in all cases. Further progress in diagnosis, prevention and treatment will likely require a better understanding of the underlying neural and cognitive mechanisms that lead directly to the symptoms and impairments of ADHD, and can be targeted by treatment interventions.

**Fig. 1:**
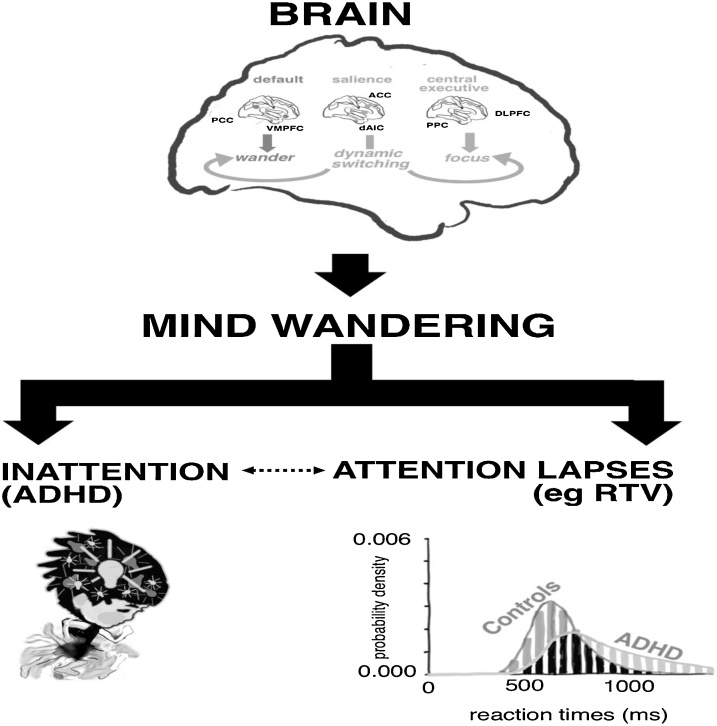
Visualisation of the linear relationship between neural activity, mind wandering (MW), inattentive symptoms and attentional lapses in the MW hypothesis. The top, central image represents the three neural networks underlying excessive and spontaneous mind wandering in ADHD. The key hubs of the default mode network are the posterior cingulate cortex, and ventromedial prefrontal cortex, associated with active mind wandering. The central executive network includes dorsolateral prefrontal cortex, and posterior parietal cortex, which are active during cognitively demanding tasks and serve as a marker of task focus. The salience network involves the anterior cingulate cortex, and anterior insula, linked to the regulation of the interaction between the default mode and central executive network. The bottom, left image represents inattentive symptoms in ADHD. The bottom, right image represents the greater variability in the distribution of reaction time scores in ADHD.

Here we propose a novel approach that focuses on a measurable component of ADHD psychopathology: excessive, spontaneous mind wandering (MW) (Mowlem et al., 2016). We put forth a new hypothesis for ADHD (see Fig. 1) in which aberrant regulation within the default mode networks, and between default mode and executive control networks, leads to spontaneous MW, which in turn leads to symptoms and impairments of ADHD, and may also underlie some of the cognitive performance deficits seen in ADHD. This is an alternative to the usual model, which views measures of cognitive function, such as sustained attention and inhibitory control deficits, as intermediate endophenotypes on the pathway from genes to behaviour (Castellanos and Tannock, 2002; Rommelse et al., 2008).

We propose this as a promising new avenue for research as MW has been linked to ADHD and ADHD-associated impairments, and unlike ADHD symptoms such as inattention, MW can be measured using a range of direct and indirect measures. These include rating scale state and trait measures, experience sampling in daily life, experience sampling during experimental paradigms, and the neural correlates of MW. Potentially these may provide new clinical and neural biomarkers of ADHD that could provide new insights into the neurobiological basis of ADHD, which can be used for diagnosis and prediction and monitoring of treatment effects, and could lead to novel treatments targeting the regulation of MW in ADHD.

## 2 ADHD, mind wandering and the default mode network

### 2.1 What is mind wandering?

Mind wandering (MW) occurs when one’s mind drifts away from the primary task and focuses on internal, task-unrelated thoughts and images. MW is a universal experience that represents up to 50% of daily thinking time (Smallwood and Schooler, 2015). While some forms of MW can be beneficial to individuals (e.g. strategic thinking about a grant proposal while driving a car), other forms can be detrimental (e.g. spontaneous uncontrolled thoughts that interfere with tasks such as listening to a lecture). These two types of MW have been referred to as deliberate and spontaneous, respectively, and are thought to reflect a different balance of regulatory processes on internal self-generated thought (Christoff et al., 2016; Seli et al., 2015). Spontaneous MW, detrimental to performance, has been proposed as a mechanism explaining many of the symptoms and impairments of ADHD (Mowlem et al., 2016; Seli et al., 2015) believed to reflect dysfunctional connectivity between the brain’s default mode network (DMN) and executive control networks (Fox et al., 2015; Sripada et al., 2014).

### 2.2 Spontaneous mind wandering is associated with ADHD

The first study of MW in ADHD was conducted using an experience sampling technique to measure on-task and off-task thoughts during a simple attention task (Shaw and Giambra, 1993).The frequency of task-unrelated thoughts was found to be increased in college students with a childhood history of ADHD diagnosis, compared to controls. Among the controls, male and female groups that reported high levels of childhood ADHD symptoms also demonstrated more task-unrelated thoughts than controls reporting low levels of childhood ADHD symptoms.

A further study, using the MW Deliberate and Spontaneous scales (Carriere et al., 2013) found that a group who had been diagnosed with ADHD showed more spontaneous than deliberate MW (Seli et al., 2015). They further showed that spontaneous MW (but not deliberate MW) was significantly correlated with ADHD symptom severity. In another study using an adult community sample, a composite index of ADHD symptoms was positively correlated with a composite index of MW derived from experience sampling data of task-unrelated thoughts during a lab session, and daily life (Franklin et al., 2017). Furthermore, ADHD symptoms were related to MW episodes that were detrimental to the task at hand, and a sub-clinical group with high ADHD symptom scores had disruptive MW episodes that impaired daily-life function. ADHD symptomatology was also positively correlated with a lack of awareness of engaging in MW. In this study, lacking awareness of MW mediated between ADHD symptoms and impairment, suggesting that increasing awareness of MW in ADHD might lead to functional improvements (Franklin et al., 2017).

In our own studies, we developed a clinical scale reflecting ADHD patient reports of excessive spontaneous MW (Mowlem et al., 2016). The 12-item Mind Excessively Wandering Scale (MEWS) captures three characteristics of MW in ADHD: thoughts constantly on the go, thoughts flitting from one topic to another, and multiple thoughts at the same time. Exploratory factor analysis found the 12-item MEWS to be unidimensional, with a single factor explaining 70% of the variance (Mowlem et al., 2016). Two independent samples revealed significantly elevated ratings of MW in ADHD, and that MW successfully discriminated between cases and controls to a similar extent as rating scale measures of DSM-IV ADHD symptoms (sensitivity and specificity around 0.90 in both studies). MEWS scores were also correlated strongly and positively with measures of inattention (r = .77), hyperactivity/impulsivity (r = .69) and ADHD-related impairment (r = 0.81). Furthermore, repeated analysis over a 6-month period showed moderate to high covariation of change in MEWS scores with change in inattention (r = .53), hyperactivity/impulsivity (r = .31) and impairment (r = .62). Regarding impairment, MEWS scores were the strongest predictor of functional impairment, followed by inattention and hyperactivity/impulsivity, indicating the clinical relevance of MW as a predictor of impairment in daily life.

Overall, these findings suggest that asking about the subjective experience of MW alone, provides a better prediction of ADHD associated impairment than the traditional ADHD inattention and hyperactive/impulsive symptoms. Moreover, as we will discuss, MW may reflect the primary deficit arising directly from dysregulated neural network activity in ADHD that underpins the symptoms and impairments currently used to define ADHD.

### 2.3 Mind wandering and the default mode network

The neural basis of MW has generated considerable interest since 2006 (Callard et al., 2013). The DMN has been implicated as a potential source of self-generated thoughts unrelated to external goal-directed tasks. The DMN reflects a network of interacting brain regions (i.e. medial prefrontal cortex, posterior cingulate cortex and medial temporal regions) which show correlated neural activation, most active during the resting state, when the person is awake but in a daydreaming or MW state. The network can be conceptualised as switching off during external goal-directed tasks, and switching on when there are internal self-generated thoughts (Buckner and Vincent, 2007).

Among the first investigations of MW-associated neural activity were two functional magnetic resonance imaging (fMRI) studies using tasks with low cognitive demand (McKiernan et al., 2006), or highly practiced cognitive tasks (Mason et al., 2007), during which episodes of MW were frequent. These studies found a strong correlation between the reduced deactivation of the DMN during on-task conditions and frequency of subjectively-reported MW. The MW-associated neural activity patterns were in stark contrast with those seen during novel or high cognitive demand conditions, when MW was less frequent. McKiernan et al. (2006) conducted three different cognitive tasks under three levels of task difficulty, making a total of nine different task/difficulty conditions, each associated with a different frequency of task-unrelated thoughts. They reported that 81% of the variance in the frequency of task-unrelated thoughts was explained by task-induced deactivation of the DMN, which is remarkably high for an association between a direct measure of brain function using fMRI, and subjective reports of a mental phenomenon. Similarly, Mason et al. (2007) found activation in DMN regions during episodes of MW, including the precuneus.

A limitation of these early studies was that the frequency of task-unrelated thoughts was measured outside the scanning sessions. To provide a more direct investigation of neural activity during periods of MW, Christoff et al. (2009) used in-scanner experience-sampling probes to identify periods of task-related and task-unrelated thoughts during a sustained attention task. The experience-sampling probes asked two questions: “Where was your attention focused just before the probe – on task or off task?”, and “How aware were you of where your attention was focused – aware or unaware?”. The strongest activation was seen in two normally anti-correlated networks (default mode and executive control networks) during MW episodes, and even stronger during “unaware” compared to “aware” MW.

The finding of co-activation of both executive and default networks during periods of task-unrelated thoughts in control subjects was subsequently confirmed by meta-analysis of fMRI studies (Fox et al., 2015). Examining 24 functional neuroimaging studies of spontaneous thought processes, meta-analysis using activation likelihood estimation (ALE) found that both DMN regions (medial prefrontal cortex, posterior cingulate cortex, medial temporal lobe, bilateral inferior parietal lobule), and non-DMN regions (rostrolateral prefrontal cortex, dorsal anterior cingulate cortex, insula, temporopolar cortex, secondary somatosensory cortex, and lingual gyrus) were consistently recruited during periods of spontaneous thoughts. They concluded that in addition to DMN activity, fronto-parietal network (FPN) and other non-DMN regions also played a central role in the neuroscience of MW and other forms of spontaneous thoughts.

However, not all studies have reported co-activation of DMN and executive control regions during periods of MW. Andrews-Hanna et al. (2010) introduced longer delays between stimuli. These delays induced MW which was most strongly correlated with DMN hubs rather than co-activation of both DMN and executive control networks. Likewise, in another study, task-unrelated thoughts were associated with the highest level of DMN (medial prefrontal cortex) activation compared to intermediate levels of activation for external distraction and task-related inferences, and the lowest DMN activity during periods of on-task thoughts (Stawarczyk et al., 2011a). Further evidence comes from studies focusing on the role of executive control mechanisms in MW.

### 2.4 Mind wandering and executive control

Overall the findings discussed above confirm the association of DMN activity with the frequency of task-unrelated thoughts. However, the role of executive control networks may depend on task conditions or type of MW and other forms of spontaneous thought. Typically, executive control and DMN function in an anti-correlated manner. Early work showed increased activation in executive control and decreased activation in DMN regions with increasing attentional demands, and vice versa (Fox et al., 2005). Likewise, attenuated DMN activity during periods of on-task was linked with poor adjustment to attentional demands, or attentional lapses (Weissman et al., 2006). Therefore, task-unrelated thoughts are expected to be associated with anti-correlation (or reduced co-activation) between executive control and DMN regions.

However, as discussed, fMRI research has been inconclusive on the role of executive control on MW, which sparked off a debate. One argument is that MW results from a failure in executive control to prevent automatic task-unrelated thoughts from becoming conscious (McVay and Kane, 2012). For example, the emergence and increased frequency of MW under high demand conditions is associated with deficits in executive control, including working memory capacity and response inhibition (McVay and Kane, 2012). An opposing argument states that executive control is required to maintain personally salient task-unrelated thoughts (i.e. strategic/deliberate MW) during low cognitive demand conditions, and is associated with reduced MW under high cognitive demand conditions to prevent performance decrements (Smallwood, 2010; Smallwood and Schooler, 2006). In line with this view, MW increased linearly with time-on-task in low demand vigilance paradigms (Randall et al., 2014; Thomson et al., 2015) and is more frequent in practiced compared to novel tasks (Mason et al., 2007).

To investigate the relative role of different neural networks, an activation likelihood estimation (ALE) meta-analysis was conducted on different types of internal thoughts, including experimentally directed episodic future thinking, and spontaneous MW (Stawarczyk and D’argembeau, 2015). The results showed that while these domains of internal thought activated a common set of brain regions within the default network (e.g. medial prefrontal cortex); regions supporting executive control processes (e.g. dorsolateral prefrontal cortex) were also recruited to a lesser extent during undirected MW than during directed episodic future thinking. Thus, different types of internally generated thoughts may recruit varying levels of executive control.

To address the various findings, recent models of MW better reflect the complexity of internally generated thoughts. They clarify that MW is not a unitary construct, but rather an umbrella term that captures different types of MW experiences in the general population. A key conceptual paper proposes a dynamic framework in which MW is understood as a subtype of spontaneous-thought phenomena that also includes creative thought and daydreaming (Christoff et al., 2016). The authors propose a dimension of deliberate constraints related to executive control activity, with unconstrained daydreaming at one end, and constrained goal-directed thought at the other. Creative thought is proposed to reflect a more constrained form of spontaneous thought that is under greater executive control than daydreaming or MW, but less than goal-directed thoughts. They further propose a second dimension of automatic constraints on content of thought, which is weakest for common forms of daydreaming, and strongest for mental phenomenon such as ruminations and obsessions. Christoff et al. (2016) proposed that under their model, ADHD would be reflected by a problem with excessive variability in thought movement, with low deliberate constraints (excessive MW and dream-like thoughts) and low automatic constraints (thoughts that flit from one topic to another).

### 2.5 ADHD is associated with deficient regulation of DMN activity

Resting state connectivity studies of ADHD in children and adults have examined interactions between the DMN and executive control network, as well as connectivity within the DMN itself. These studies consistently find that anti-correlation between the executive control (fronto-parietal) network and DMN is attenuated, and that resting state connectivity within the DMN itself is reduced (Posner et al., 2014). ADHD is associated with hyperactivation (deficient deactivation) of the DMN in task compared to resting state conditions (Fassbender et al., 2009; Helps et al., 2010; Liddle et al., 2011; Peterson et al., 2009).

The observation of DMN abnormalities in ADHD led to a hypothesis known as the DMN interference model (Sonuga-Barke and Castellanos, 2007). This model proposed that increased very low frequency oscillations (0.01 - 0.1 Hz) and synchronization within the DMN, which usually attenuate during goal-directed tasks, persist and interfere with task-specific neural processes, leading to lapses in attention and performance deficits (Sonuga-Barke and Castellanos, 2007). Reduced DMN deactivation from rest to task, and suppressed DMN activity (up-regulation) at the transition to rest conditions, suggest inadequate neural switching in response to changes in context in ADHD compared to controls (Liddle et al., 2011; Sidlauskaite et al., 2016). Consistent with this hypothesis, Liddle et al. (2011) found that failure to deactivate DMN regions during a low-salient (slow, boring, unrewarded) inhibition task requiring sustained attention, was reversed by methylphenidate in children with ADHD. In a further study both improvement of inattentive symptoms and normalisation of very low electroencephalography (EEG) frequencies as well as omission errors followed the use of methylphenidate (Cooper et al., 2014).

Related to the default mode interference hypothesis, it has also been proposed that activation of DMN hubs will depend on motivation, cognitive load, attentional demands and individual state regulation/capacity (Stawarczyk et al., 2011a). This is in line with the cognitive-energetic model which proposes that the efficiency of task performance in ADHD is determined by the interplay of basic cognitive processes (e.g. stimulus encoding, memory search, binary decision and motor preparation), and the availability of these processes related to arousal and activation levels and is further modulated by interplay with executive control functions (Sergeant, 2005). Both the DMN interference and cognitive-energetic models point to an inability to adapt neural network activity to changing task demands.

The DMN interference hypothesis also proposed that a certain threshold of DMN activity needs to be reached before DMN interference occurs (Sonuga-Barke and Castellanos, 2007). In relation to our model of MW in ADHD, we propose that this critical threshold of DMN activity is linked to excessive spontaneous MW, which leads to the inattentive symptoms of ADHD and detrimental effects on daily-life function. Cognitive performance deficits associated with ADHD may be a direct result of internal distractibility secondary to excessive MW, or may be secondary to the direct effects of high default mode activity interfering with task-dependent neural functions. We further propose that this critical threshold of DMN activity may not be reached when there is a continuous co-activation of DMN and executive control regions associated with deliberate/strategic forms of MW. For example, individuals without a clinical diagnosis of ADHD may more readily co-activate DMN and executive control regions, and engage in deliberate rather than uncontrolled/excessive forms of spontaneous MW. Previous authors have also proposed that reduced deactivation of the DMN during task performance may explain MW and interference with task positive processes, leading to the symptoms and impairments of ADHD (Liddle et al., 2011; Mowlem et al., 2016; Posner et al., 2014). However, at the time of writing this has yet to be formally evaluated.

## 3 A comparative analysis of ADHD and mind wandering

So far, we have outlined studies that link ADHD to MW, MW to DMN activity, and DMN activity to ADHD. These studies raise the possibility that deficient regulation of DMN activity leads to excessive spontaneous MW in individuals with ADHD, which might underpin the inattentive symptoms of ADHD and deficits in cognitive task performance. Further support for this hypothesis comes from several observations that draw parallels between processes that underlie the regulation of MW in neurotypical controls, and processes found to be deficient in ADHD. These parallels include: (1) context regulation of MW in controls, and deficient context regulation of neural activity in ADHD; (2) perceptual decoupling of somatosensory processing during MW, and in ADHD; (3) sensitivity of MW and MW-associated neural processes to task salience and rewards; (4) impairments in cognitive task performance, and function in daily life. Below we outline these areas in more detail.

### 3.1 Context regulation of mind wandering

The context regulation hypothesis states that an adequate capacity to self-regulate mind wandering within a context will reduce a potential negative impact on the primary task performance (Smallwood and Andrews-Hanna, 2013). Context regulation of MW is characterized by adaptation of neural processes and frequency of MW to changing task demands. In community samples, MW frequency is higher during low perceptual and low cognitive demand conditions (Filler and Giambra, 1973; Giambra and Grodsky, 1989) and lower under more cognitively demanding conditions, such as tasks with high working memory demands (Antrobus et al., 1966). As discussed above Mason et al. (2007) manipulated the frequency of MW by varying task cognitive-demands, and correlated MW frequency with DMN task induced deactivations. Similar findings have been replicated by others (Forster and Lavie, 2009; Levinson et al., 2012; McVay and Kane, 2012; Metcalfe and Xu, 2016; Ruby et al., 2013; Rummel and Boywitt, 2014; Smallwood et al., 2007; Xu and Metcalfe, 2016). Related to these findings, shifts from task-related to task-unrelated thoughts are observed during low cognitive demand tasks (Stawarczyk et al., 2011b; Thomson et al., 2014).

Regarding the potential role of executive control and working memory capacity, individuals with both low and high working memory capacity report greater levels of MW under low cognitive demand conditions (Kane et al., 2007). However, under high demand conditions, task-unrelated thoughts are greater in individuals with low working memory capacity, compared to those with higher working memory capacity (Kam and Handy, 2014; Kane et al., 2007; Unsworth and Robison, 2016). This effect is consistent with the role of executive control in the appropriate regulation of MW during cognitively demanding tasks.

Furthermore, higher working memory capacity predicted greater frequency of task-unrelated thoughts in a low demand breath-awareness task (Levinson et al., 2012) but fewer errors (regarded as a behavioural index of less frequent MW) in a high demand 3-back working memory task (Rummel and Boywitt, 2014). Similarly, the level of linguistic expertise (reflecting executive control and working memory capacity) determined the frequency of MW since individuals with high levels of linguistic ability showed more frequent task-unrelated thoughts for easy items, compared to individuals with low to medium linguistic ability (Xu and Metcalfe, 2016). Thus, both working memory capacity and linguistic capacity had a moderating effect on the level of MW under low and high demand conditions.

In summary, these findings indicate that higher working memory/executive control capacity moderate frequency of MW according to task demands. In general, greater working memory capacity is predictive of more MW during low demand conditions, whereas it is predictive of less MW during high demand conditions.

### 3.2 Context regulation in ADHD

Context regulation of MW in ADHD has yet to be investigated. However, there is consistent evidence for deficient neural adaptation to task demands in ADHD compared to controls, with deficient upregulation of executive control regions, accompanied by deficient deactivation of the DMN. Thus, the neural networks that show deficient context regulation in ADHD are the same networks associated with context regulation of MW in neurotypical controls.

For example, deficient context regulation was seen in ADHD compared to controls using electroencephalography EEG recordings during the Flanker Task. This task contrasts a low cognitive demand (no conflict) with a high cognitive demand (conflict) condition. In contrast to the ADHD group, controls showed a significantly greater increase in frontocentral theta amplitude and decreased phase variability (which correlated with less reaction time variability, RTV) in response to the higher cognitive demands of the conflict condition (McLoughlin et al., 2009, 2014). Low phase variability over trials is thought to reflect an adaptive mechanism to maintain stable neural processing of a stimulus (Makeig et al., 2004; Papenberg et al., 2013). Therefore, the relationship between increased theta phase variability and RTV in ADHD points to a reduced ability to maintain efficient neural adaptation and cognitive performance over trials with increasing cognitive demands.

Similarly, there was greater RTV in ADHD compared to controls, under high demand (very fast or very slow event rates), compared to low demand (medium event rates) conditions (Metin et al., 2016) reflecting deficient behavioural adaptation of task performance to changing task demands. Deficient context regulation of neural (EEG) activity in ADHD is also seen in the transition from rest to task conditions (Rommel et al., 2016; Skirrow et al., 2015); a finding that was reversed in response to methylphenidate (Skirrow et al., 2015).

fMRI studies also demonstrate deficient context regulation in ADHD. Compared to controls, ADHD is associated with under-activation of the FPN (left dorsolateral prefrontal cortex) during on-task conditions (Castellanos and Proal, 2012; Cortese et al., 2012) and reduced deactivation of the DMN (medial prefrontal cortex) when transitioning from rest to task (Valera et al., 2010). Attenuated deactivation and hyperactivation within the DMN in ADHD compared to controls was also observed with time-on-task during a sustained attention task, in response to high working memory load, and with longer inter-stimulus delays (Christakou et al., 2013; Liddle et al., 2011; Metin et al., 2015; Paloyelis et al., 2007; van Rooij et al., 2015).

Taken together, the evidence from cognitive-EEG and fMRI studies points to a reduced ability to modulate task positive and negative neural processes in ADHD. Since the networks involved are the same as those linked to context regulation in neurotypical controls, deficient context regulation in individuals with ADHD may explain the high frequency of task-unrelated thoughts. This hypothesis has yet to be formally tested.

### 3.3 Perceptual decoupling in mind wandering

A key characteristic of MW is the association with attenuated somatosensory processing, referred to as perceptual decoupling. This means that during periods of MW there is a reduced somatosensory response to sensory stimuli (Schooler et al., 2011; Smallwood et al., 2013). One hypothesis is that perceptual decoupling explains the co-activation of the FPN and DMN, during low demand conditions; reflecting active executive control over attention to disengage from perceptual input, so as to enable mental processing of personal goals (Smallwood et al., 2012). In line with this, MW has been linked to anti-correlation and lack of synchronisation between sensory cortices and DMN (Christoff, 2012; Kirschner et al., 2012) and a positive correlation between sensory cortices and FPN hubs during on-task conditions. A novel concept suggests that the depth of perceptual decoupling might be able to distinguish between spontaneous and deliberate MW (Seli et al., 2015).

EEG research has consistently reported that event-related potential (ERP) components (P1), markers of early visual information processing within 100 ms, are attenuated during periods of task-unrelated compared to task-related thoughts (Baird et al., 2014; Broadway et al., 2015; Kam and Handy, 2014). Phase-locking factor analyses reflect phase synchrony of a particular frequency at a particular time across multiple trials of an event (Tallon-Baudry et al., 1996). Episodes of MW were further linked to a lower phase-locking factor in theta within 50–150 ms following a visual stimulus reflecting a neural state of perceptual decoupling during MW (Baird et al., 2014).

Cortical source activity analyses during a visual Sustained Attention to Response Task (SART) also confirm the attenuation of early visual information processing, during periods of MW (Kirschner et al., 2012). In this study, there was also deficient intra-regional (occipital cortex) and inter-regional (visual cortex and right medial temporal lobe) connectivity when attention was focused on internal thoughts (Kirschner et al., 2012). In contrast, during periods when the participants were focused on the visual task, there was greater inter-regional connectivity between the visual cortex and task-positive regions including the anterior/posterior cingulate, orbitofrontal cortex and posterior parietal gyrus. Similarly, MW was linked to both an increase in occipital and parieto-central theta and fronto-central delta power and a decrease in occipital alpha and frontal lateral beta power (Braboszcz and Delorme, 2011).

Collectively, these findings suggest a switch from active cognitive processing to MW, which is facilitated by a state of perceptual decoupling, or detachment of perception from attention. Consequently, a reduced P1 amplitude is regarded as a marker of perceptual decoupling during episodes of MW.

### 3.4 Perceptual decoupling in ADHD

Somatosensory responses are far less studied in ADHD, and the association between sensory decoupling and ADHD is not well established. Furthermore, there have been no studies that directly investigate the relationship of perceptual decoupling to periods of MW in ADHD. Yet, the few studies that have focused on early sensory processing in ADHD find deficits that are similar to those seen during periods of MW in neurotypical controls.

Initial reports found decreased slow frequency fluctuations within the left sensorimotor cortex (Yu-Feng et al., 2007) and suppression of visual ERP amplitudes during cognitive-performance tasks in children with ADHD compared to controls (Steger et al., 2000). Using magnetoencephalography (MEG), adults with ADHD showed reduced event-related desynchronization in the alpha band, and synchronisation in the beta bands, in primary and secondary somatosensory cortices in response to median nerve stimulation (Dockstader et al., 2008). A similar attenuated cortical sensory response was found in children with ADHD, which improved following successful treatment with methylphenidate (Lee et al., 2005).

Using ERP, a larger P1 (100 ms post-stimulus) amplitude has been seen in children with ADHD compared to controls; a finding that was interpreted as a compensatory mechanism in the absence of performance differences (Kóbor et al., 2015; Shahaf et al., 2012). In contrast, when children with ADHD made more omission errors than controls, P1 amplitude was significantly reduced (Nazari et al., 2010). At the time of writing, preliminary findings from our group support this result. Using the SART, we found a reduced P1 amplitude in 33 adults with ADHD compared to 30 controls (p < 0.02), which was associated with trait measures of inattention, and MW measured using the MEWS as a state measure of excessive MW in ADHD (Bozhilova et al., unpublished data). We further found that in the ADHD cases there was a reduced P1 amplitude prior to errors compared to correct responses (p < 0.001). Under the assumption that MW will be higher prior to error than non-error responses, these findings suggest that somatosensory processing deficits could be linked to excessive MW in ADHD. This hypothesis has yet to be formally tested.

### 3.5 The effects of salience and reward on mind wandering

Several studies find that the frequency of MW is related to task salience or incentives designed to increase motivation. This is not surprising, since almost everyone finds it easier to remain focused on tasks that are inherently interesting, compared to mundane or boring tasks. A high degree of task-related motivation and interest were both associated with lower frequency of MW during the SART (Seli et al., 2015, 2016) as well as better information retention on a film comprehension task (Kopp et al., 2016).

A related phenomenon is the response to unexpected infrequent stimuli, which tend to be of high salience in auditory oddball paradigms. Infrequent and deviant auditory stimuli were associated with an increased mismatch negativity amplitude, which is a marker of attention allocation to these stimuli, during episodes of task focus compared to a decreased amplitude during MW (Braboszcz and Delorme, 2011). Therefore, MW is linked to poorer attentional engagement with task-salient stimuli. An automatic, momentary coupling of attention and perception for visually salient stimuli (coloured no-go targets) during MW, also occurred in a variation of the Go/No-Go task (Smallwood, 2013) again suggesting switch away from MW.

In another study, monetary incentives increased the number of reports of self-caught MW compared to conditions that were unrewarded (Zedelius et al., 2015). The incentive for accuracy of MW self-report was associated with less probe-caught MW in the absence of greater overall MW (Zedelius et al., 2015). This last finding indicates that reward is likely to increase awareness of MW. Overall, these findings suggest that the effects of task salience and reward have the potential to reduce MW and MW-associated neural activity.

### 3.6 The effects of salience and reward on ADHD

There are numerous examples of enhanced sensitivity to the effects of task salience and rewards in children and adults with ADHD. Here we discuss some of the most pertinent studies related to MW-associated task performance and neural activity.

In a key fMRI investigation, a sample of children with ADHD and controls completed a Go/No-go task under low- and high-incentive conditions (Liddle et al., 2011). In the low-incentive condition (with low rewards linked to task performance), there was attenuated DMN deactivation in the ADHD group compared to controls. When higher rewards were introduced to increase the salience of the task, DMN deactivation normalised to the same level as controls. Methylphenidate was also found to have the same effect on DMN deactivation in the ADHD group, as increasing the salience of the task through rewards. The authors concluded that both methylphenidate and enhanced salience normalised DMN deactivation and suggested that this had an impact on inattention.

In a series of publications from Kuntsi and colleagues, fast-rewarded (high incentive) conditions compared to slow-unrewarded (low incentive) conditions on tasks requiring sustained attention reduced or abolished ADHD case-control differences for arousal measures using skin conductance (James et al., 2016), RTV (Andreou et al., 2007; Cheung et al., 2017; Kuntsi et al., 2009; Tye et al., 2016) and omission errors (Uebel et al., 2010), but not commission errors (Kuntsi et al., 2009).

Based on the findings that enhancing task saliency alters neural responses, task performance and frequency of MW, we hypothesise that adequate rewards will modulate the frequency of MW in both neurotypical and ADHD populations. In particular, a decrease in MW will lead to better early attentional orienting in ADHD, or successful detection of visual information early on at presentation. Despite similar effects on DMN activity of task saliency (reward) and stimulants in ADHD (Liddle et al., 2011), different mechanisms could be involved. For instance, rewards may moderate the degree of early visual information detection via interactions between the DMN and visual cortex. In contrast, stimulants might lead to changes in MW by altering interactions between large-scale networks: for example, FPN and DMN; DMN and ventral attention network; and DMN and the salience network. Providing both reward and stimulants reduce the frequency of MW and/or facilitate a more controlled form of MW, a combination of both might further enhance treatment effects. Further research is needed to investigate the effects of reward and stimulants on MW in ADHD and mechanisms involved in both ADHD and controls.

### 3.7 Cognitive performance and daily life impairments associated with mind wandering

MW has an adverse impact on both cognitive task performance and daily life in control populations. Measured performance deficits associated with MW include greater stimuli-response error rates (Forster and Lavie, 2009; Stawarczyk et al., 2011b), slower reaction times (Smallwood et al., 2013; Stawarczyk et al., 2011b) reduced accuracy of response (Kam and Handy, 2014; McVay and Kane, 2009; Rummel and Boywitt, 2014; Smallwood et al., 2008; Thomson et al., 2014; Unsworth and Robison, 2016; Xu and Metcalfe, 2016) and greater mean error percentage (Thomson et al., 2015).

Similarly, attenuation in the amplitude of ERPs reflecting late cognitive analysis of stimuli (e.g. attentional resource allocation, response execution/preparation and attentional orienting) has been observed during periods of MW. Examples include reduced N400, mismatch negativity (Braboszcz and Delorme, 2011) and P3 amplitudes (Barron et al., 2011; Riby et al., 2008; Smallwood et al., 2007; Villena-González et al., 2016) as well as greater central negativity (N2) (Riby et al., 2008). Further, frequent MW and poorer effort were related to poorer accuracy (Brown and Harkins, 2016; Seli et al., 2016).

MW has also been associated with several daily life impairments, including deficits in reading comprehension (Mooneyham and Schooler, 2013), negative mood (Smallwood et al., 2009a, b; Smallwood and O’Connor, 2011), poorer learning (Metcalfe and Xu, 2016; Xu and Metcalfe, 2016), higher number of car accidents (Galéra et al., 2012) less cautious driving (Yanko and Spalek, 2013), poorer academic performance, life-satisfaction, self-esteem and greater stress (Mrazek et al., 2013).

### 3.8 Cognitive performance and daily life impairments associated with ADHD

Similar impairments in cognitive task performance and daily life associated with MW are also seen in ADHD. Examples include attentional orienting, performance monitoring, response preparation and inhibitory processes; manifesting in increased error rates, reaction time variability and attenuation in Cue-P3 and No-Go N2 and P3 amplitudes (Albrecht et al., 2014; Cheung et al., 2017; McLoughlin et al., 2009, 2010; Michelini et al., 2016a; Tye et al., 2014; Uebel et al., 2010).

ADHD is also associated with impairments in daily life including poorer psychosocial, educational and global function (Asherson et al., 2012; Pitts et al., 2015). Individuals with ADHD compared to controls are more prone to car accidents (Vaa, 2014), academic underachievement (Fischer et al., 1990; Weyandt and DuPaul, 2008), reading comprehension difficulties (Ghelani et al., 2004; Miller et al., 2013; Stern and Shalev, 2013) and low self-esteem (Shaw-Zirt et al., 2005).

In summary, the findings on impairment show that both ADHD and MW in non-ADHD controls appear to be linked to similar performance deficits. This is consistent with our MW hypothesis for ADHD since we propose that episodes of MW in controls and excessive spontaneous MW in individuals with ADHD lead to similar functional and cognitive impairments. Potentially, excessive spontaneous MW in ADHD can be expected to lead to typical impairments experienced by people with ADHD in their daily lives. Examples could include problems following a conversation, reading and watching a film; maintaining a coherent train of thought for problem solving and holding thoughts in mind; difficulties falling asleep due to constant mental restlessness associated with excessive MW; and feeling exhausted by the mental effort required to sustain focus on daily activities. Further research is required to investigate the extent to which impairment seen in individuals with ADHD can be explained by excessive, spontaneous MW.

### 3.9 Identifying causal processes

As discussed extensively in this review, deficient deactivation of the DMN during cognitive task performance has been consistently reported in ADHD. However, ADHD is associated with a wide range of cognitive and neural deficits, anyone of which could be contributing to the generation of ADHD symptoms, and be potential targets for treatment. Indeed, the range of deficits associated with ADHD has led many to argue that the neuropathological basis for ADHD is highly heterogeneous (Coghill et al., 2005; Faraone, 2015).

Key additional processes highlighted in the literature include: deficits in the dorsal attention network of dorsolateral prefrontal cortex, basal ganglia and parietal regions during tasks of selective and sustained attention (Hart et al., 2013); inhibitory control deficits supported by fMRI studies of reduced activation in key regions of inhibitory control (Plichta and Scheres, 2014). Furthermore stimulants, the first line treatment for ADHD, has been shown to have effects on several of these processes (Rubia et al., 2014, 2009; Smith et al., 2013).

Despite the findings linking various neural functions to ADHD, it remains unclear which of these play a direct causal role in the generation of the inattentive and hyperactive/impulsive symptoms currently used to define ADHD. This is a critical point, because numerous non-causal associations are expected to arise through the process of pleiotropy, in which shared genetic (and environmental) risks can lead to multiple different phenotypic outcomes at the level of brain structure, function, cognitive performance and behaviour. Yet, only a subset of the associated neural deficits may reflect underlying causal processes in ADHD (Kendler and Neale, 2010). There are several approaches that can be used to address this problem (Kendler and Neale, 2010), including conducting mediation analyses with experimental trial data, or longitudinal outcome data, or taking advantage of genetic data to test for causal associations using Mendelian randomisation (Sheehan et al., 2011).

### 3.10 Remission and persistence of ADHD

In relation to ADHD, important new insights have come from longitudinal outcome studies that investigate the cognitive and neural correlates of remission and persistence in ADHD (Franke, 2015). A central question in causal models of ADHD has been the separation of executive control measures (e.g. inhibition and working memory) from preparation-vigilance measures. Earlier work showed that measures reflecting these processes are both associated with ADHD in children, but are genetically largely uncorrelated, reflecting distinct aetiological pathways (Kuntsi et al., 2010, Kuntsi et al., 2014).

Regarding developmental change, in a 6-year longitudinal outcome study of children with DSM-IV ADHD, adolescents and young adults who no longer met ADHD criterial (ADHD remitters), but not those who still had ADHD (ADHD persisters), showed a similar profile to controls without ADHD on cognitive and neural measures. These measures indicated response preparation, attention and vigilance processes (RTV, omission errors, delta activity, errors in low-conflict conditions and contingent negative variation) and were significantly correlated to the severity of inattention at outcome. In contrast, executive control measures of working memory, and inhibitory processes did not distinguish in persisters and remitters, and did not correlate with ADHD symptoms at outcome; including commission errors, digit span backwards, and ERP activity of inhibitory control (No-go P3) and conflict monitoring (N2) (Cheung et al., 2016; Michelini et al., 2016b).

Similarly, in another follow-up study there was no an association between ADHD remission and improvements in executive functioning (Biederman et al., 2009), interference control (Pazvantoğlu et al., 2012), and response inhibition (McAuley et al., 2014). Working memory impairments (e.g. reduced caudate activation) in young adults diagnosed with ADHD in adolescence compared to controls have also been observed regardless of whether they still met an ADHD diagnosis (Roman-Urrestarazu et al., 2016).

A recent study reported increased resting-state fMRI connectivity in ADHD remitters compared to controls in the executive control network, with intermediate connectivity profiles in persisters (Francx et al., 2015). Two other connectivity studies (Michelini et al., 2017; Mattfeld et al., 2014) also indicated that connectivity within the executive control network or during executive tasks may not distinguish between ADHD persisters and remitters. Both persisters and remitters showed an increased EEG connectivity during executive control (Michelini et al., 2017) and reduced negative functional correlation between medial (DMN hub) and dorsolateral prefrontal cortex (FPN hub) during resting state fMRI (Mattfeld et al., 2014).

However, in a resting study fMRI, only ADHD persisters showed reduced positive functional correlation between DMN hubs (posterior cingulate and medial prefrontal cortices) (Mattfeld et al., 2014). Reduced posterior cingulate cortex and medial prefrontal cortex connectivity (i.e. DMN intra-connectivity) may therefore be a neurobiological marker of persistence of ADHD (Uchida et al., 2015).

From these findings we conclude that, potentially, DMN activity and preparation-vigilance processes (associated with MW) may have direct aetiological significance in ADHD since they track the symptoms of ADHD during child to adult development. In contrast, deficits in executive control functions appear to dissociate from the clinical course of ADHD symptoms during development and are therefore less likely to play a direct causal role in the ongoing generation of symptoms. Since, the neural processes tracking the clinical disorder are closely aligned to those thought to underpin MW, we propose that targeting the regulation of MW or MW-associated neural dysfunctions could potentially lead to reductions in ADHD symptoms and remission of the disorder.

## 4 Developmental perspective on mind wandering in ADHD

### 4.1 Mind wandering and typical development of functional networks

Finally, we provide a developmental perspective on the neural processes linked to MW, since the frequency and impact of MW may vary throughout development. “Small-world” properties represent quantifiable metrics of topographic properties of large-scale/global brain organisation (Achard et al., 2006). These properties are present by the age of 7, and resemble functional brain organisation in young adults (Supekar et al., 2009). The two major hubs of the DMN (PCC and mPFC; also neural correlates of MW), are visible very early on, with DMN formation completed by 2 years of age (Gao et al., 2009). However, formation of the DMN is thought to precede functional specialisation for self-referential cognition and mentalising such as mind wandering (Gao et al., 2009).

An increase in long-range connections, as well as a decrease in short-range connections (segregation) and strengthening of within-network interactions (integration), are thought to govern functional brain development and functional specialisation (Di Martino et al., 2014; Kelly et al., 2008; Power et al., 2010; Rubia, 2013; Rubia et al., 2009). The childhood to adolescent years (ages 7–15) are a particularly sensitive period for such functional brain network development (Mak et al., 2017), which is the same age that ADHD symptoms and impairments often emerge (Asherson et al., 2016). We also propose MW is likely to become more frequent during daily life, or even emerge. During this period greater integration develops between DMN and somatosensory regions, contrasting their complete segregation in adulthood. Additionally, early adolescence is marked by a similar degree of DMN deactivation contrasting less task-related deactivations in somatosensory regions compared to adults (Thomason et al., 2008). This deactivation pattern was linked to better task performance in childhood (Thomason et al., 2008), suggesting that the recruitment of somatosensory areas might be a compensatory strategy during high cognitive demand task conditions in children. In summary, while children exhibit a DMN activity similar to adults, the system does still undergo important changes between middle childhood and young adulthood. Therefore, frequency and type of MW may vary with age as function of DMN development.

Within-network integration of DMN hubs (Fair et al., 2008) and segregation from other networks is considered to be a good marker of brain maturation (Dosenbach et al., 2010; Rubia et al., 2009), and better than executive control networks whose functional development is less affected by age (Sato et al., 2014). However, executive control networks also show a pattern of strengthening of intra-network and weakening of inter-network connections from childhood to middle adulthood (Fair et al., 2007). These developmental changes are reflected in functional improvement, or greater adaptive control and greater working memory capacity with age (Fair et al., 2007). Similarly, a pattern of increasing anti-correlation between large-scale networks (DMN and FPN) and task-related DMN deactivations is likely to support improved regulation of both executive control and MW (Fair et al., 2008). Potentially, a maturational delay in the functional specialisation of these networks could result in a disrupted regulation of executive control and MW.

### 4.2 Mind wandering and “Maturational lag hypothesis” of brain development in ADHD

Related to the findings on typical functional brain development, El-Sayed et al. (2003) proposed a maturational lag hypothesis. The hypothesis suggests that a persistent maturational lag in functional brain development might become a sustained functional abnormality leading to symptoms and impairments of ADHD. We further propose that abnormalities in resting-state functional connectivity resulting from co-activation of functionally related brain regions (Power et al., 2010) may lead to the self-generation of excessive, spontaneous and context-independent thoughts, typical of MW, which are externalised as inattentive behaviours over the lifespan. Consistent with this view, recent work in ADHD has shown a maturational lag in major large-scale brain networks, especially within-network integration (DMN and FPN) and interactions between default mode, frontoparietal, ventral attention and salience networks (Sripada et al., 2014). A recent review also summarised findings for decreased synchrony/connectivity between the two major DMN hubs in ADHD (Castellanos and Aoki, 2016). The lagged maturation was associated with DMN interference, poor performance (e.g. greater reaction time variability) during cognitively demanding tasks, and was proportionate to the severity of inattention (Sripada et al., 2014).

With regard to the development of MW in ADHD, to date there has been only one published study, which compared the frequency of MW in children and adults with ADHD (Van den Driessche et al., 2017). Using an experience sampling method, similar frequencies of “mind blanking” (MW without awareness of the content) were seen in 6-12-year-old children and young adults with ADHD. While the authors found no case-control performance differences on the SART, medication-naïve children and adults reported twice as much mind blanking, but fewer episodes of task focus and MW with awareness than controls. We propose that individuals with ADHD in this study tended to report mind blanking rather than MW due to the lack of a coherent reportable content. When comparing a group of children with ADHD treated with methylphenidate with drug naïve children and controls, there was reduced frequency of mind blanking to the level of controls; although the treated group still had a greater frequency of MW with awareness of content than controls (Van den Driessche et al., 2017). Medication was therefore proposed to allow access to awareness of MW. The authors (Van den Driessche et al., 2017) further hypothesised that this effect could be due to restoration of executive resources. These findings suggest similar abnormalities in the frequency of MW-related measures in ADHD during both childhood and young to middle adulthood.

Overall, the developmental studies suggest that understanding the cortical maturation of key networks leading to aware/unaware and spontaneous/deliberate forms of MW, may be important to understanding the onset, course and development of ADHD.

## 5 Discussion

This review sets out to draw parallels between MW and ADHD, and guide new insights into ADHD psychopathology. The theoretical conceptualisations outlined are designed to set the scene for further hypothesis testing. The basis for this work is the observation that a spontaneous and poorly controlled type of MW is strongly associated with ADHD, and ADHD-related impairments. Currently, there are only a small number of studies that measure MW in ADHD, and none that simultaneously measure ADHD, MW and neural functions associated with both ADHD and MW. The relationship of MW to ADHD symptoms such as inattention is therefore not yet well understood. We therefore set out to summarise what is known about the links between ADHD, MW and their neural correlates.

At the level of neural networks, there are strong parallels in the association of both ADHD and MW with DMN activity during task conditions. Reduced deactivation of DMN activity during tasks requiring sustained attention is associated with the frequency of MW in controls, and is also associated with ADHD compared to controls. Since ADHD is also linked to a specific pattern of spontaneous and poorly controlled MW, a simple hypothesis can be proposed that the normal neural processes that regulate MW are disturbed in ADHD, leading to excessive levels of spontaneous and uncontrolled MW. Furthermore, cognitive and functional impairments that are associated with periods of MW in controls are comparable to the range of impairments associated with ADHD, suggesting that excessive spontaneous MW may underpin functional deficits in ADHD.

In support of our hypothesis we draw on evidence that the usual processes regulating MW are disrupted in ADHD. First, we see that the usual context regulation of DMN and executive control network activity to increasing task demands is associated with MW, and deficient in ADHD. Furthermore, deficient regulation of these processes appears to be reversed by stimulant medications used to treat ADHD. Second, we see that the processes associated with both MW and ADHD are sensitive to the salience of task conditions. In ADHD increasing the salience of task conditions may “normalise” performance and associated neural processes. Finally, we see that both MW and ADHD may be associated with sensory decoupling such as the reduced early P1 response of the occipital cortex to a visual stimulus. We therefore propose that by understanding the processes of normal regulation of MW in the general population, we can identify with more precision aberrant processes in ADHD.

One question that arises from this model is whether measures of MW can be considered distinct from the inattentive symptoms of ADHD. For example, measures of MW may merely reflect alternative measures of the same underlying construct. Currently, there is almost no evaluation of the incremental information provided by measures of MW, although we did report that a rating scale measure of MW was an independent predictor of impairment in ADHD after controlling for ADHD inattention and hyperactivity/impulsivity symptoms (Mowlem et al., 2016). Further research is therefore required to addresses this question. However, since many of the findings related to ADHD also apply to measures of MW, it is a reasonable hypothesis that one reflects the other.

If there is a very close relationship of MW with ADHD-inattention, this would open new avenues for research. MW has the advantage that it can be measured using simple rating scale trait measures, but also experience sampling data in daily life (considered to be more objective reflections of the mental state); and experience sampling during experimental paradigms such as sustained attention tasks, which have been used to investigate the neural correlates of MW in control samples. Measures of MW may also be less dependent than the inattentive symptoms of ADHD on behavioural adaptation, which is influenced by learnt coping strategies, so perhaps a better reflection of the underlying neural condition.

The model we propose views the inattentive symptoms of ADHD as outcomes of MW (i.e. internal distractibility), and MW in ADHD may be a direct outcome of altered neural regulation of internal thought processes. Cognitive performance deficits seen in ADHD may also be a direct outcome of MW. We propose that dysregulated neural functions (DMN overactivity during task conditions for example), are reflected in excessive spontaneous MW, and that such internal distractibility explains behavioural symptoms such as losing track during conversations, avoiding tasks that require sustained attention, not completing tasks, and misplacing things.

Further research is required to investigate heterogeneity the range of internally generated thoughts, and understand the regulatory processes involved. A key question is to fully understand the differences in studies which find that episodes of MW are associated with both reduced or absent (Kucyi et al., 2016; Stawarczyk and D’argembeau, 2015) and present (Christoff, 2012; Spreng et al., 2010) co-recruitment of DMN and executive control network regions. In our view, this likely reflects that MW is a multidimensional construct and different types of MW recruit the executive control network differentially.

Spontaneous, unaware (‘zone-off’) MW was associated with decreased functional connectivity (Golchert et al., 2017) and a negative functional correlation (Gruberger et al., 2011) between the DMN and FPN. This pattern contrasts with increased functional connectivity between these two networks during deliberate MW (Christoff et al., 2016). Potentially, both spontaneous and deliberate MW might involve the co-activation of both networks initially. This co-activation allows both deliberate MW and task performance under low demand to be maintained, as well as the controlled and adequate shift to task focus under high demand. Alternatively, this co-activation might also reflect an inability to suppress the DMN activity during on-task conditions. Therefore, constant engagement in spontaneous MW might represent a neural state of overly active DMN hubs during on-task, and hypoconnectivity within the entire DMN at rest. The uncontrolled and spontaneous occurrences of MW will then result in poor context regulation.

In our review, we identified three existing fMRI accounts proposing specific dynamics of MW. The dynamic framework (Mittner et al., 2016) proposes a greater activation of the dorsal attentional network during on-task that transitions to increasing connectivity/activity within the DMN during an off-focused state (selecting between returning to the task or entering a state of MW). Finally, if task-unrelated thoughts appear more salient, an individual enters a state of MW marked by reduced functional connectivity between large-scale networks (Mittner et al., 2016). The other two fMRI accounts speculate on initial recruitment of the right medial temporal lobe, or limbic regions (Golchert et al., 2017) and later FPN (Fox et al., 2015) during MW. However, the dynamics of MW remain poorly understood (Smallwood et al., 2011).

Future research could focus on the use of EEG, which can capture fast and covert cognitive processes such as MW. The increased use of state-of-the-art EEG approaches that provide improved source localisation of EEG signals, combined with EEG’s millisecond temporal resolution, has already demonstrated their better potential to explain the dynamics of MW compared to more traditional scalp-level EEG analyses (Braboszcz and Delorme, 2011; Kirschner et al., 2012).

## 6 Specific predictions from the mind wandering hypothesis for ADHD

The MW hypothesis proposes that altered interaction between the four large scale networks (DMN, executive control network, salience network and visual network), and that deficient DMN deactivation during task activities will lead to excessive spontaneous MW, lacking in coherence and topic stability, which in turn will lead to ADHD symptomatology.

To investigate this hypothesis, future work could apply experimental designs that have been successfully applied to manipulate and measure MW and measures the neural correlates of MW in controls samples, to ADHD case-control studies, or by investigating correlations to dimensional measures of ADHD symptoms. Examples include sustained attention and inhibitory control tasks, varying cognitive load by using manipulations such as altering the stimulus presentation rate (Christakou et al., 2013) or introducing a working memory component (Baird et al., 2014). This would enable investigation of context regulation and sensory decoupling in relation to ADHD. A further manipulation would be to the comparison of high and low reward conditions, to investigate the effects of salience (Liddle et al., 2011).

Inferring causal directions may be difficult because of the close temporal timing of neural, cognitive performance and MW events if they covary strongly with each other. One approach to infer causal relationships is to use treatment interventions such as stimulant mediation, to bring about change in the various outcome measures (Kendler and Neale, 2010). Then the causal relationships between these can be modelled. Mindfulness training is another intervention that is thought to act on the regulatory processes involve in MW and ADHD (Mrazek et al., 2014). Recent studies suggest effects of mindfulness training that are potentially comparable to those seen for ADHD medications (Hepark et al., 2015).

To guide future work, we outline specific predictions derived from this hypothesis.

### 6.1 Perceptual decoupling

We propose that individuals with ADHD will experience greater perceptual decoupling driven by the proposed ADHD-specific MW, compared to controls during long-lasting (>30 min) sustained attention paradigms. A potential neural correlate is absent or reduced inter-regional synchronisation or positive functional correlation between DMN and visual networks. Using EEG, reduced mean P1 and N1 amplitudes will reflect inefficient early perceptual processes in ADHD.

### 6.2 Context regulation

We propose that individuals with ADHD will be unable to adapt to increasing cognitive (attentional, control) demands both at the neural level, and behaviourally; and that deficient context regulation of neural activity in ADHD will be related to the frequency of MW. Regarding neural correlates of these processes, we propose that a reduced positive functional connectivity between DMN and salience network will reflect the deficit in switching from rest to task, task to rest and from a low to high demand condition. Another neural correlate will be an absent change in frontal midline theta power from rest to task, hypoactivation in the DMN from task to rest and hypoconnectivity or reduced positive functional connectivity within the DMN (posterior cingulate cortex and medial prefrontal cortex) during rest.

### 6.3 Behavioural correlates of DMN and FPN dysregulation

We propose that variable reaction times will stem from MW and serve as a behavioural correlate of reduced negative functional correlation (or even disconnection) between default mode and executive control network and hyperactivation within the DMN during cognitive paradigms.

The neural inefficiency in the large-scale networks will manifest in increased phase inconsistency/variability of parietal or frontal theta in response to task-related stimuli, which will coincide with excessively frequent episodes of MW.

Changes in the content and context of MW is likely to reflect differential recruitment of DMN regions (Stawarczyk and D’argembeau, 2015). For instance, depressive thoughts might result in greater connectivity between the core DMN and salience network, and a weaker connectivity between these two networks with the FPN and the medial temporal lobe (DMN hub). Excessive MW was correlated with the highest degree of DMN activation, suggesting that overactivity of the DMN might reflect the frequency rate of MW (Kucyi et al., 2016). Therefore, MW in ADHD will be characterised by hyper-connectivity within both right medial temporal lobe and right medial prefrontal cortex (DMN hubs) during on-task.

## 7 Conclusion

Converging evidence indicates that both MW (Kucyi et al., 2016) and ADHD (Sidlauskaite et al., 2016) are linked to DMN regulation and regulation of the interaction between DMN and FPN. In particular, ADHD is associated with both within DMN and between DMN-FPN dysregulation. These neural effects are present in ADHD independent of age, clinical characteristics and type of task (Cortese et al., 2012; Plichta and Scheres, 2014), supporting the MW hypothesis. A dysfunctional and later absent interaction between the four major networks (DMN, executive control network, salience network and visual network) is proposed to underlie different aspects of cognitive and behavioural impairment associated with MW in ADHD.

Future studies should focus on understanding whether completely different neural processes underlie MW in ADHD compared to controls, or there is simply neural attenuation in ADHD. Understanding context regulation requires the use of conditions varying in cognitive demand. Another necessary study is to measure effects of reward, or whether reward ‘normalsies' MW in ADHD compared to controls. Importantly, validation across different MW measures and conceptualization of different types of MW will enable the investigation of neural activity underlying specific types of MW.

## Conflicts of interest

Professor Jonna Kuntsi has given talks at educational events sponsored by Medice: all funds are received by King’s College London and used for studies of ADHD. Kings College London research support account for Professor Philip Asherson received honoraria for consultancy to Shire, Flynn-Pharma, Eli-Lilly, Novartis, Lundbeck and Medice; educational/research awards from Shire, Lilly, Novartis, Flynn Pharma, Vifor Pharma, GW Pharma and QbTech; speaker at sponsored events for Shire, Lilly, Novartis, Medice and Flynn Pharma.
